# Normative Database of Spatiotemporal Gait Metrics Across Age Groups: An Observational Case–Control Study

**DOI:** 10.3390/s25020581

**Published:** 2025-01-20

**Authors:** Lianne Mobbs, Vinuja Fernando, R. Dineth Fonseka, Pragadesh Natarajan, Monish Maharaj, Ralph J. Mobbs

**Affiliations:** 1 Wearable and Gait Assessment Research (WAGAR) Group, Prince of Wales Private Hospital, Randwick, NSW 2031, Australia; lianne@koinis.com.au (L.M.);; 2 Faculty of Psychology, University of New South Wales (UNSW), Sydney, NSW 2033, Australia; 3 NeuroSpine Surgery Research Group (NSURG), Sydney, NSW 2031, Australia; 4 Neuro Spine Clinic, Prince of Wales Private Hospital, 320-346 Barker St., Randwick, NSW 2031, Australia; 5 Faculty of Medicine, University of New South Wales (UNSW), Sydney, NSW 2033, Australia

**Keywords:** spatiotemporal gait analysis, normative database, IMU, inertial measurement units

## Abstract

Introduction: Gait analysis is a vital tool in the assessment of human movement and has been widely used in clinical settings to identify potential abnormalities in individuals. However, there is a lack of consensus on the normative values for gait metrics in large populations. The primary objective of this study is to establish a normative database of spatiotemporal gait metrics across various age groups, contributing to a broader understanding of human gait dynamics. By doing so, we aim to enhance the clinical utility of gait analysis in diagnosing and managing health conditions. Methods: We conducted an observational case–control study involving 313 healthy participants. The MetaMotionC IMU by Mbientlab Inc., equipped with a triaxial accelerometer, gyroscope, and magnetometer, was used to capture gait data. The IMU was placed at the sternal angle of each participant to ensure optimal data capture during a 50 m walk along a flat, unobstructed pathway. Data were collected through a Bluetooth connection to a smartphone running a custom-developed application and subsequently analysed using IMUGaitPY, a specialised version of the GaitPY Python package. Results: The data showed that gait speeds decrease with ageing for males and females. The fastest gait speed is observed in the 41–50 age group at 1.35 ± 0.23 m/s. Males consistently exhibit faster gait speeds than females across all age groups. Step length and cadence do not have clear trends with ageing. Gait speed and step length increase consistently with height, with the tallest group (191–200 cm) walking at an average speed of 1.49 ± 0.12 m/s, with an average step length of 0.91 ± 0.05 m. Cadence, however, decreases with increasing height, with the tallest group taking 103.52 ± 5.04 steps/min on average. Conclusions: This study has established a comprehensive normative database for the spatiotemporal gait metrics of gait speed, step length, and cadence, highlighting the complexities of gait dynamics across age and sex groups and the influence of height. Our findings offer valuable reference points for clinicians to distinguish between healthy and pathological gait patterns, facilitating early detection and intervention for gait-related disorders. Moreover, this database enhances the clinical utility of gait analysis, supporting more objective diagnoses and assessments of therapeutic interventions. The normative database provides a valuable reference future research and clinical practice. It enables a more nuanced understanding of how gait evolves with age, gender, and physical stature, thus informing the development of targeted interventions to maintain mobility and prevent falls in older adults. Despite potential selection bias and the cross-sectional nature of the study, the insights gained provide a solid foundation for further longitudinal studies and diverse sampling to validate and expand upon these findings.

## 1. Introduction

i. Objectives

The present study is an observational case–control study of 313 healthy participants to systematically collect and analyse spatiotemporal gait metrics. Using a single-point inertial measurement unit (IMU), the study aims to establish a normative database for gait metrics across varied age groups, contributing to the broader understanding of human gait dynamics.

ii. Gait as an objective measure of health

Gait is an objective reflection of an individual’s health status, functionality, and the interplay between musculoskeletal and neurological systems [1]. The quantification and analysis of gait parameters, such as speed, step length, and cadence, offer valuable insights into the physiological changes associated with ageing, the impact of various health conditions, and the effectiveness of therapeutic interventions. The quantification of gait dynamics often relies on stereophotogrammetric techniques, which provide a robust theoretical framework for reconstructing skeletal kinematics and analyzing joint variables [2].

Deterioration of gait velocity has been shown in the literature to be linked to the likelihood of future hospitalisation, falls risk, cognitive decline, mental health deterioration, and mortality in general [3,4,5]. Debate remains on the application of various gait metrics, and although there has been some uptake in various health circumstances, its integration is not standardised in clinical practice.

iii. Spatiotemporal Gait Analysis

This paper focuses on spatiotemporal gait analysis, examining metrics such as gait velocity, cadence, and step length through accelerometry and wearable devices. Schwesig et al. (2011) conducted a landmark study with 1860 participants (aged 5–100 years), using IMUs to measure key gait parameters like step length, cadence, and walking speed. Their findings highlighted the scalability of wearable sensors for large-scale gait analysis [6]. Building on this foundation, our study addresses critical gaps by expanding normative datasets to include diverse age and height groups.

iv. Clinical Utility of Spatiotemporal Gait Data

Table 1 highlights the diagnostic potential of spatiotemporal gait data by summarising findings that compare pathological metrics with healthy populations. While previous studies often normalise parameters by height, this can obscure individual differences crucial for clinical diagnostics. Non-normalised data offer raw measurements that better identify deviations from normative patterns, particularly for specific gait abnormalities. By reporting both normalised and non-normalised datasets, this study ensures broader applicability for clinical and research use, contingent on a reliable baseline for comparison.

v. The need for a normative database

A comprehensive normative spatiotemporal database remains absent in the literature, as evidenced by gaps in Table 1. Most existing studies rely on lab-based optical stereophotogrammetry, which, while accurate, fails to capture ‘free-living gait’ due to effects like the Hawthorne and “white coat” phenomena. While stereophotogrammetry provides detailed insights into skeletal movement and joint kinematics [2], its application in free-living gait scenarios is limited by the controlled environment required. Additionally, research often focuses on narrow age ranges (e.g., 70+), as seen in Hollman et al. [7], leaving other groups underrepresented. This study addresses these gaps by providing datasets that span diverse age and height groups, offering clarity and applicability for clinical and research use. Unlike the pathological data summarised in Table 1, our findings focus solely on healthy, normative gait, expanding the scope of previous work to better serve a broad array of applications.

The present study bridges the gap in the literature by compiling a comprehensive gait database with the aid of inertial measurement units (IMUs). IMUs are wearable single-point devices with an accelerometer, magnetometer, and a gyroscope. Measurements made with IMUs have not only been validated against laboratory analysis techniques (r > 0.83) but can also capture free-living gait in the community [8]. This empowers clinicians and researchers alike to make conclusions that are both externally valid and generalisable. However, it is important to acknowledge that while IMUs offer the advantage of portability and the ability to collect free-living data, they are not without limitations. Recent studies have shown that IMUs can be prone to noise and biases, providing less accurate estimations of human motion compared to camera-based motion capture systems [9]. This may result in cumulative errors when deriving gait parameters, particularly when integrating acceleration data. Nonetheless, the small, inexpensive, and unobtrusive nature of IMUs has enabled the collection of gait data across diverse age groups, facilitating the development of a comprehensive database of gait characteristics [8,10,11]. This paper enhances our understanding of how gait evolves with age and provides essential reference points for clinicians to detect deviations from normal gait patterns, aiding in the early detection of gait-related disorders. Moreover, the paper considers the effects of height and highlights the intrinsic relationship between gait patterns and physical stature, ultimately advancing the field of spatiotemporal gait analysis in medical settings.

**Table 1:** Summary of gait alteration in various pathological conditions.

Gait Velocity	Cadence	Stride Length	Stride Time	Stride Time Variability	Double Support Time
Parkinson’s Disease [12,13,14,15,16,17,18,19]					
−(8–11)%	−6%	−(7–17)%	+(6–8)%	+76%	+24%
Lumbar Disc Herniation [20]					
−76%	−66%	-	-	-	+53%
Chronic Mechanical Lower Back Pain [20,21,22]					
−(13–26)%	−19%	-	-	-	+(14–16)%
Lumbar Spinal Stenosis [23,24,25,26,27]					
−(12–37)%	−(10–14)%	-	-	-	-
Depression [28]					
−3%	-	-	-	-	+0.03%
Hip Osteoarthritis [29]					
−14%	−5%	−10%	-	-	+13%
COPD [30,31]					
−7%	−(7–13)%	-	+15%	-	+(16–17)%

## 2. Materials and Methods

i. Ethics

Ethics approval was obtained from the South Eastern Sydney Local Health District (LNR/16/POWH/535). All patients provided written informed consent for the procedure and access to data on their ongoing clinical evaluation and radiological outcomes.

ii. Study Population

The study’s cohort consisted of 313 normative subjects. Participant demographic and baseline characteristics are summarized in Table 2. Spatiotemporal metric trends by age are detailed in Table 3, providing insights into variations in gait parameters such as gait speed, cadence, and step length across different age groups.

Participants were excluded if they were under 18 years of age, had a body mass index (BMI) over 45, were unable to walk at least 50 m independently, were pregnant, or had any medical conditions known to alter gait patterns, such as stroke, lumbar spinal stenosis, multiple sclerosis, or significant degenerative and/or rheumatological conditions affecting the hip, knee, and spine. However, in the age categories 21–30, 31–40, and 51–60, individuals with minor or self-reported balance issues were included as they did not meet the exclusion criteria for significant balance impairment. These balance issues were controlled for during analysis to ensure they did not unduly affect the normative values established in the study.

iii. Data collection

**Figure 1:**
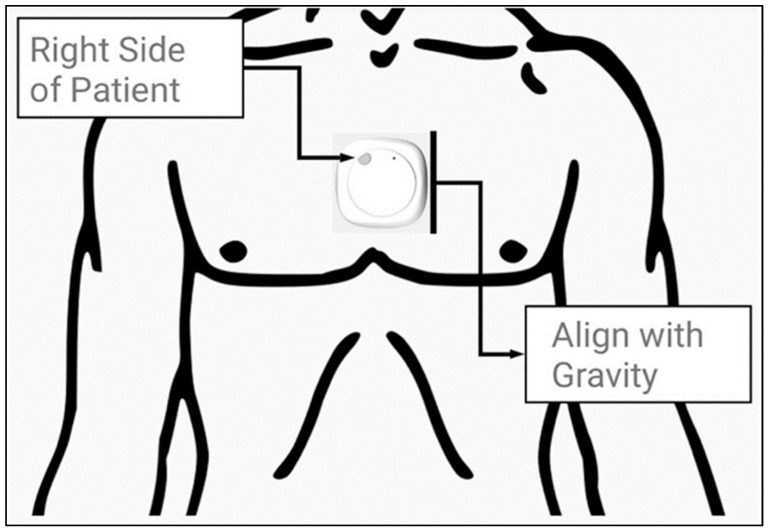
The MetaMotionC© (MMC) inertial measurement unit (IMU) developed by Mbientlab Inc. pictured as it was fitted on the sternal angle of patients. Figure taken from Natarajan et al. [33].

Participants provided informed consent and underwent structured interviews to collect demographic data (Table 2). Gait data were recorded using the MetaMotionC IMU (MbientLab Inc., San Francisco, CA, USA), equipped with a 16-bit triaxial accelerometer (100 Hz), gyroscope (100 Hz), and magnetometer (0.3 μT at 25 Hz). The device setup and placement are shown in Figure 1. The IMU was placed at the sternal angle for optimal data capture during a 50 m walk on a flat, unobstructed pathway. Calibration ensured correct sensor orientation, and walks were performed unobserved to simulate natural conditions. While device calibration ensured measurement accuracy, the specific accuracy of this methodology remains unvalidated. The IMUGaitPY algorithm, adapted from GaitPY [32], demonstrates high reliability in prior studies but is subject to typical IMU-related errors (e.g., percentage errors in gait parameters) [8]. Future research should address these limitations. Studies such as Washabaugh et al. [8] demonstrate high validity (e.g., IMU reliability r > 0.83), supporting the robustness of IMU-based gait data in free-living conditions.

Data capture was facilitated through a Bluetooth™ connection to an Android™ smartphone running the custom-developed IMU Gait Recorder application. The raw data collected were then processed using IMUGaitPY, a specialised version custom-coded by the WAGAR Group (Sydney, Australia) of the open-source GaitPY Python package Version 1.6.1, adapted by Czech and Patel [1] for enhanced gait metric analysis. This software was instrumental in extracting the relevant spatiotemporal metrics from the collected data, with further methodological details provided in Appendix A.

## 3. Data Analysis

Inferential and descriptive analyses were conducted, with normality assessed using the Shapiro–Wilk and Kolmogorov–Smirnov tests. For normally distributed data, the mean and standard deviation were calculated to summarise central tendency and variability.

Non-normal data underwent rank transformation to support hypothesis testing on age- and height-related variations. Outliers were excluded using the interquartile range (IQR) method, ensuring data integrity while preserving variability. ANOVA was employed to test hypotheses on parameter dependence, given its effectiveness in comparing means across multiple groups. Non-normality is a common issue in gait analysis due to the inherent variability and non-linearity of gait patterns. Gait metric parameters such as step length, stride length, and gait speed often show skewed distributions with outliers, making them challenging to analyse using traditional statistical methods that assume normality.

Consequently, the use of this method transformed the original gait dataset into a more normally distributed form, hence improving the validity and interpretability of our statistical analyses.

The choice of rank transformation was made because of its ability to handle non-normal data while preserving the ordinal relationship among the data points. Unlike other transformations such as logarithmic or square root transformations, which may alter the interpretation of the data, rank transformation retains the rank order of the data, making sure that the relative standing of each observation is preserved. A common method applied is the use of the inverse normal cumulative distribution function, or the probit function, to transform ranks into standard normal deviates, resulting in transformed values that approximate a normal distribution.

To assess the efficacy of the rank transformation, distributional properties of the original and transformed data were compared. Measures of skewness and kurtosis were calculated for both sets of data. It was found that rank transformation led to a significant reduction in skewness and kurtosis, which indicates a closer approximation to normality. Visual inspection histograms and QQ plots of the original and transformed data further confirmed the improvement in normality after transformation. In conclusion, rank transformation is a useful tool for dealing with non-normal data in gait analysis. Transforming the original spatiotemporal data into a more normally distributed form improved the validity and interpretability of our statistical analyses.

All statistical analyses were conducted using IBM SPSS software, version 27.0.

## 4. Results

i. Study Population

After cleaning our data, we excluded data pertaining to 33 normative subjects from our analysis. Specifically, 17 subjects were removed because of incomplete demographic information, 4 subjects were excluded due to errors attributed to an IMUGaitPy software bug, and an additional 12 subjects were eliminated due to the presence of excessive noise in their data, as indicated by their aberrant spatiotemporal parameters. After these exclusions, the study’s cohort was refined to consist of 313 normative subjects.

ii. Spatiotemporal Metric trends by age

**Figure 2:**
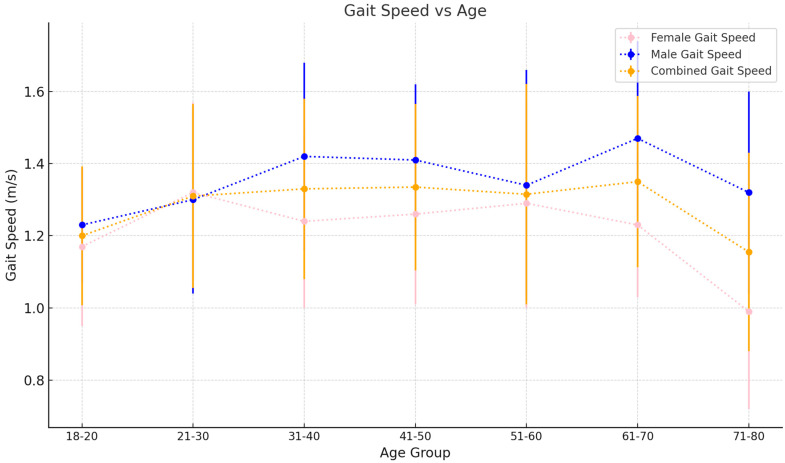
Gait speed as it varies with age. Values plotted are the mean±SD for each 10-year age group.

**Figure 3:**
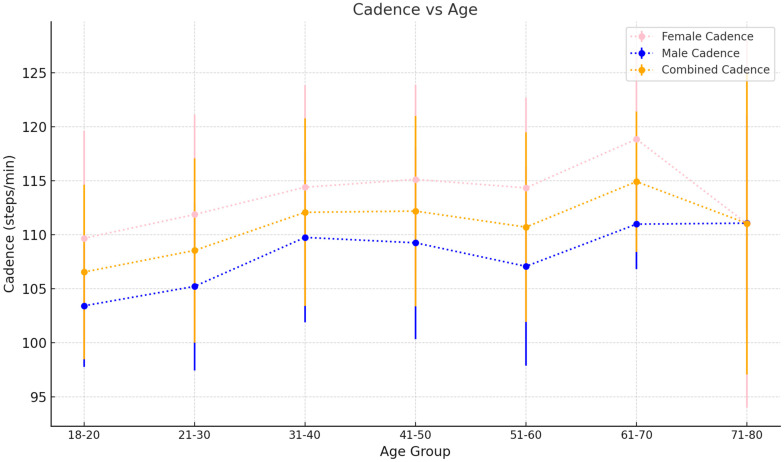
Cadence as it varies with age. Values plotted are the mean±SD for each 10-year age group.

**Figure 4:**
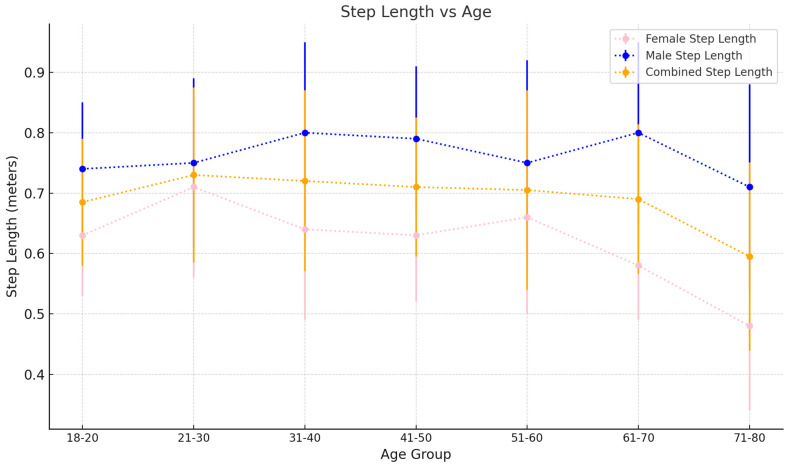
Step length as it varies with age. Values plotted are the mean±SD for each 10-year age group.

Mean gait speed increases steadily from 1.21 ± 0.19 m/s in the 18–20 age bracket to 1.36 ± 0.24 m/s in the 41–50 age group before steadily decreasing again to 1.16 ± 0.31 m/s in the 71–80 age group (see Figure 2). Cadence increases from 105.8 ± 8.02 steps/min in the 18–20 age bracket to 111.9 ± 8.87 steps/min in the 31–40 age group (see Figure 3). This remains steady until the 51–60 age group, where mean cadence is 110.06 ± 9.48 steps/min before sharply increasing to 115.92 ± 7.86 steps/min in the 61–70 age group. However, this decreases again in the 71–80 age group to 111.06 ± 12.50 steps/min. Mean step length starts at 0.69 ± 0.12 m in the 18–20 age bracket and increases to 0.73 ± 0.14 m in the 21–30 age group (see Figure 4). This decreases slightly in the next three 10-year age backets, to a value of 0.72 ± 0.17 m in the 51–60 age bracket. This value decreases again for the 61–70 and 71–80 age groups at 0.67 ± 0.16 m and 0.60 ± 0.19 m, respectively.

iii. Spatiotemporal Metric trends by age and gender (females)

In females, mean gait speed increases from 1.17±0.22 m/s in the 18–20 age bracket to 1.32±0.25 m/s in the 21–30 age bracket. This value decreases to 1.24±0.26 m/s in the 31–40 age group before increasing again to 1.28±0.25 m/s and 1.29±0.30 m/s in the 41–50 and 51–60 age groups, respectively. Mean gait speeds then see a more noticeable decrease in the 61–70 and 71–80 age groups at 1.23±0.20 m/s and 0.99±0.27 m/s, respectively. Cadence increases from 109.7±9.95 steps/min in the 18–20 age group to 118.8±8.21 steps/min in the 61–70 age group before finally decreasing to 111.03±17.0 steps/min in the 71–80 age group. Step length does not follow a consistent trend (Table 4). Similarly, in males, gait speed and cadence trends are summarized in Table 5. Mean step length values are similar for the 18–20, 31–40, 41–50, and 51–60 age groups. There is an increase in the 21–30 age group at 0.71±0.15 m and lower values at 61–70 (0.58±0.10 m) and 71–80 age groups (0.48±0.14 m).

iv. Spatiotemporal Metric trends by age and gender (males)

In males, gait speed sees an increase from the 21–30 age group (1.23±0.16 m/s) to the 31–40 age group (1.43±0.27 m/s). Speeds in this range are sustained in the 61–70 age group as well. Notably, there is a decrease (1.34±0.32 m/s) in the 51–60 age group in males, which does not fit the overall trend. In comparison to females, males exhibit faster average gait speeds, which, on average, are better sustained as they age. Females, as seen above, demonstrate a relative peak in gait speed at the 31–40 age group, before demonstrating an overall decreasing trend as they age. Cadence in males is consistently lower than that of females in all 10-year age groups. Males start at a cadence of 103.42±5.63 steps/min in the 18–20 age group, and this figure increases until ages 31–40, where the cadence is 109.77±5.63 steps/min. From here, cadence is roughly consistent with ageing. Step length in males is like that in females and the whole cohort in that it does not follow a noticeable trend.

v. Spatiotemporal Metric trends by height

Gait speed generally increases with height, starting at 1.23±0.11 m/s for the 141–150 cm height bracket and culminating in a mean gait speed of 1.49±0.12 m/s for the 191–200 cm height group. Step lengths follow a similar increasing trend, although the increase is more noticeable from 0.62±0.06 m to 0.91±0.05 m for the shortest to tallest height categories, respectively. In contrast, cadence varies inversely with height starting at 116.54±6.36 steps/min for the shortest group to 103.52±5.04 steps/min in the tallest group (Table 6).

vi. Spatiotemporal Metric trends by height and gender (females)

Gait speed in females increases with height from 1.20±0.09 m/s in the 141–150 cm height group to 1.43±0.08 m/s in the 181–190 cm age group before finally decreasing to 1.21±0.10 m/s in the 191–200 cm age group. Step length increases consistently from 0.60±0.04 m in the 141–150 cm height bracket to 0.77±0.11 m in the 181–190 height bracket before decreasing again in the 191–200 cm height group to 0.60±0.05 m. Cadence follows a similar albeit inverse trend in a decreasing cadence from the shorter to the taller groups, barring the tallest group, which features a dip in cadence to 116.76±6.95 steps/min⁡ (Table 7).

vii. Spatiotemporal Metric trends by height and gender (males)

Gait speed in males decreases firstly from the 141–150 cm height group (1.40±0.00 m/s) to the 151–160 cm group before increasing steadily with height. Step length follows a similar trend, featuring an initial decrease from the shortest group to the 151–160 cm group before again steadily increasing. Cadence, in contrast, decreases consistently from the shortest to tallest group apart from the 161–170 cm group, which features a marginal increase in cadence from the 151–160 cm group (Table 8).

## 5. Discussion

The aim of this study was to establish a comprehensive normative database for spatiotemporal gait metrics across various age groups. This database serves as a crucial reference point for identifying deviations in gait patterns, which can be instrumental in clinical assessments and research. By analysing gait characteristics across a broad age range, the study provides valuable insights into the typical changes in gait with ageing, aiding in the early detection and treatment of gait-related disorders.

### 5.1. Changes with Age

i. Gait Speed

Overall, this study demonstrates that gait speeds decline with advancing age, a finding that is consistent across multiple studies in the literature [7,34,35,36,37,38,39,40]. This trend is evident in both sexes, with notable variations at different age intervals. At the early age groups (18–20 years), gait speeds are relatively lower. This increases in the 21–30 age bracket for both sexes, likely due to anthropometric developments during adolescence and early adulthood, such as increasing height [41].

Whilst females’ gait speeds start to decline after the 21–30 age group, male gait speeds continue to increase, reaching a peak in the 31–40 age group (1.43 ± 0.27 m/s). This peak is sustained in the 41–50 age group (1.41 ± 0.21 m/s), contrasting with common beliefs in the medical literature about peak physical fitness occurring between the ages of 25 and 34 [42]. This divergence suggests potential variations in fitness or health within our male sample, especially in the 30–50 age range.

The literature presents few studies which document the gait speeds in early to middle adulthood, but where it does, we see similar trends. For example, Bohannon et al. [39] shows a similar increase in average gait speed from males aged 20–29 (1.35 m/s) to ages 30–39 (1.43 m/s), which is in fact sustained until ages 50–59. Females sampled in this study, peak in average gait speed at ages 20–29, and decrease thereafter, and this is consistent with our data. Reasons for this trend could be that the males we sampled in the 30–50 age group represent a fitter and healthier subset of the wider population of 30–50-year-old males who would meet our inclusion criteria for normative gait. Conversely, it could be that the males aged 21–30 are on average less fit and healthy than the wider normative population in this age group. Alternatively, it could indeed be a combination of both reasons. It is likely that there is a large spectrum of “normal gait” within healthy males aged 21–50, and further studies sampling larger numbers of subjects are needed.

This study’s dataset also reveals an unexpected increase in female gait speeds in the 41–60 age bracket (1.28 ± 0.25 m/s), diverging from the broader literature, which suggests a steady decline post-30s [39]. Similarly, male gait speeds show an increase in the 61–70 age category (1.47 ± 0.28 m/s). These anomalies could be attributed to the possibility of our sample including healthier individuals in these age groups.

Across all age brackets, males exhibited faster average gait speeds compared to females. While females peaked in gait speed during their 20s, males not only peaked later, in their 30s, but also maintained this peak into their 40s. These results are consistent with the wider literature [34,35].

In summary, while gait speeds tend to decrease with age, our study highlights interesting variations and exceptions to this trend, emphasising the need for further research with larger and more diverse populations to gain a clearer understanding of gait speed dynamics across different age groups.

ii. Step Length

Our study reveals interesting fluctuations in step length across different age groups, which, unlike gait speed, do not follow a consistent pattern.

In the youngest age group (18–20 years), the average step length is 0.70±0.12 m for both sexes and increases to 0.74±0.14 m in the 21–30 age group. This increase could be due to height increases observed in adolescence and early adulthood, and consequently, the attainment of peak step length [43,44].

As individuals age beyond 30 through to age 60, step length does not demonstrate a clear increasing or decreasing trend. In females, the step length values are relatively stable across the 31–60 age group, fluctuating slightly but staying within a narrow range (0.64–0.67 m). This stability suggests a maintained walking efficiency in middle age. For males, a similar trend is observed, with step lengths remaining consistent in the 31–50 age groups. Khoon Lau at al. found similar fluctuations in step lengths in middle age [35]. This suggests that factors affecting step length are multifactorial and warrant more detailed investigation in future studies.

In the older age categories (61–80 years), females exhibit a decrease in step length. For females, the average step length drops to 0.59 ± 0.10 m in the 61–70 age group and further to 0.48 ± 0.14 m in the 71–80 age group. Similarly, males show a decline from 0.81 ± 0.15 m in the 61–70 age group and 0.72 ± 0.17 m in the 71–80 age group. The decline in step length is strongly consistent with the broader literature and has been attributed to various synergistic causes, be it ageing-related decreases in gait speed or even age-related musculoskeletal changes [7,37].

Overall, these findings indicate that while step length increases during early adulthood, reaching a plateau in middle age, it begins to decline in the later years. This pattern could have implications for understanding the biomechanics of ageing and for designing interventions to maintain mobility in older adults. Future studies with larger cohorts are needed to further explore these age-related changes in step length.

iii. Cadence

Our data suggest that cadence increases in early adulthood, before stabilising to a value which is sustained into advanced age (70+). In both sexes, cadence increases from 105.8±8.02 steps/min in the 18–20 age bracket to 111.87±8.87 cm in the 31–40 age group, which remains largely consistent until older age (71–80). There is an outlier in this trend where females demonstrate a sharp increase to 118.87±8.21 steps/min, which is likely due to this subset of females being fitter and healthier than the average normative population. The literature is heterogenous in its trends on cadence, with some documenting consistent cadence across adult age groups [35,45] and others demonstrating cadence decreasing with ages above 70 [7]. The reason for this is unclear as cadence is influence by a multitude of factors and can consequently show a large degree of variation in a healthy population. For example, taller individuals may take longer strides and consequently require fewer steps per unit time to achieve their comfortable walking pace, which is the manner of gait speed assessed in this study.

Consistent with this idea, cadence in males is consistently lower than that of females across all ages, and this agrees with the literature. This is explained by the fact that males are on average taller than age-matched females [46], which is reflected in males’ longer step lengths.

Overall, our study indicates that cadence increases in early life and stabilises throughout adulthood and advanced age. Importantly, the findings of this study add to the observed heterogeneity in the literature regarding how cadence changes with age and especially advanced ages (70+). Further investigation into this matter is warranted to clarify our understanding of age-related changes in walking patterns, which can consequently inform interventions aimed at maintaining mobility and preventing falls in older adults.

### 5.2. Changes with Height

To the best of the authors’ knowledge, the present study is the first of its kind to document average gait speeds, step length, and cadence systematically across all height brackets.

i. Gait Speed

**Figure 5:**
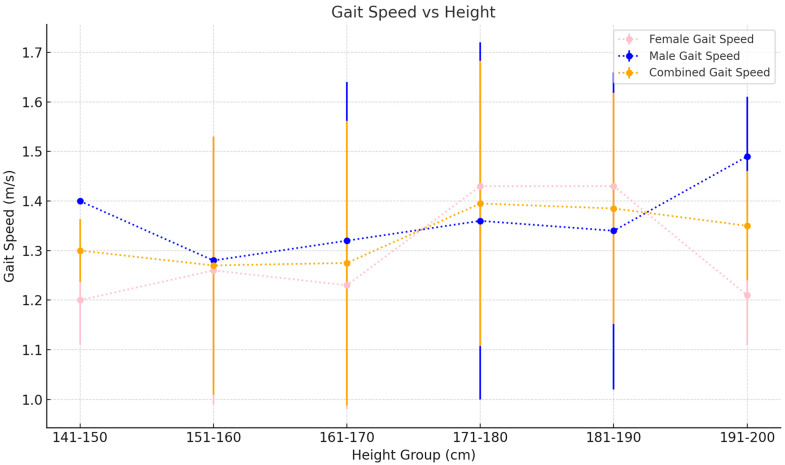
Gait speed as it varies with height. Values plotted are the mean±SD for each 10 cm height bracket.

**Figure 6:**
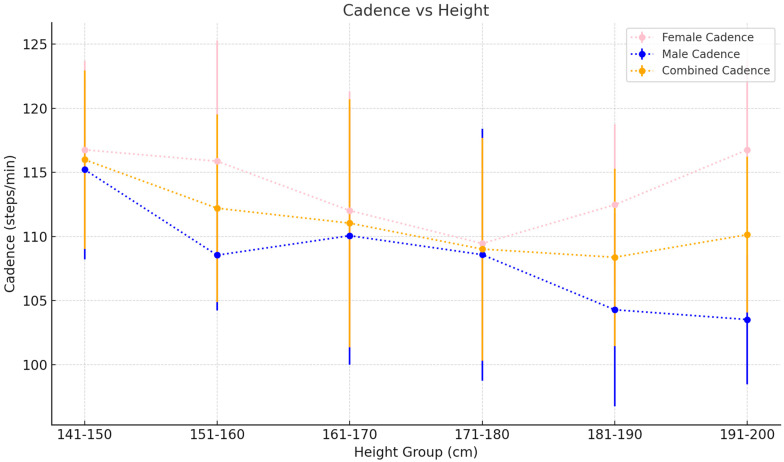
Cadence as it varies with height. Values plotted are the mean±SD for each 10 cm height bracket.

This study finds that gait speed increases steadily and predictably with increasing height in both sexes. Subjects between 141 and 150 cm in height have an average gait speed of 1.24±0.12 m/s (see Figure 5) and this increases steadily to a value of 1.49±0.12 m/s in those 191–200 cm tall (see Figure 6).

ii. Step Length

**Figure 7:**
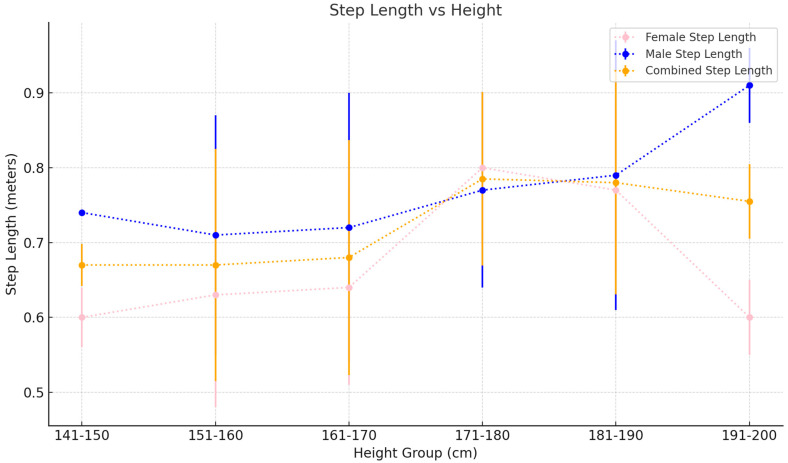
Step length as it varies with height. Values plotted are the mean±SD for each 10 cm height bracket.

Similarly, step length increases steadily and predictably with increasing height in both sexes. Subjects between 141 and 150 cm in height have an average step length of 0.62±0.07 cm, and this increases steadily to a value of 0.91±0.05 m/s in those 191–200 cm tall (see Figure 7).

These trends are understandable, as with increasing height, individuals have longer strides and hence step lengths. This is reflected in higher gait speeds, as they can cover more distance in a shorter amount of time than shorter age- and sex-matched healthy individuals. Gunasekaran et al. demonstrated a similar trend [38]. However, their data only compared gait speeds of those shorter or taller than 152 cm in females and 165 cm in males, which, critically speaking, fails to discriminate these key spatiotemporal gait metrics as they vary with height.

iii. Cadence

In contrast, cadence decreases steadily with increasing height in both sexes. Subjects between 141 and 150 cm in height have an average cadence of 116.54±06.37 steps/min, and this decreases to a value of 103.52±5.04 steps/min in those 191–200 cm tall. This trend is likely explained by the fact that taller individuals need to take less steps to cover a similar distance or achieve a similar gait speed to their shorter counterparts and hence demonstrate lower values for cadence [47].

In summary, with increasing height, we observe faster gait speeds, longer step lengths, and lower values for cadence.

### 5.3. Strengths, Limitations, and Future Directions

Our study employs a rigorous and systematic methodology for the measurement of gait parameters of individuals of diverse age and height. The dataset is comprehensive and enables extensive analyses of trends in spatiotemporal gait metrics across different groups. The insights gained from this process are valuable for clinicians and researchers alike.

Clinicians can compare their patients’ gait parameters with a normative reference range. This not only aids in distinguishing between healthy and pathological gait but also allows for the measurement of deviations from the norm, enabling the quantification of the severity of a gait-altering disease. This empowers clinicians to make more objective diagnoses and assess the effectiveness of their interventions with greater precision.

Researchers could explore the potential diagnostic and predictive applications of spatiotemporal gait data, leveraging the capabilities of modern machine learning algorithms [48]. Such algorithms have the potential to distinguish individuals with gait-altering conditions like Parkinson’s disease or lumbar spinal stenosis from healthy individuals, showcasing their diagnostic abilities [33]. Moreover, by analysing data from individuals who have experienced falls, researchers may create profiles to identify those at risk well in advance. These possibilities are just a glimpse into the innovative potential, all rooted in a robust normative database.

A key limitation of this study is the potential for selection bias. The sampling method may have led to an overrepresentation of healthier individuals, especially in certain age groups, which could skew the results. This bias could limit the generalisability of the findings to the broader population. Furthermore, the cross-sectional nature of the study restricts the ability to infer causal relationships between age, gender, and gait changes.

Future research should aim to address the current study’s limitations by incorporating a more diverse and representative sample, including individuals with varying health statuses. Longitudinal studies could provide deeper insights into how gait metrics evolve over time within individuals. Investigating additional gait characteristics and exploring the influence of lifestyle factors, such as physical activity levels, and health conditions on gait would broaden the understanding of gait dynamics. Technological advancements in gait analysis should also be leveraged to provide more nuanced and comprehensive data.

## 6. Conclusions

This study establishes one of the most comprehensive normative databases for spatiotemporal gait metrics across diverse age and height groups, providing critical tools for clinicians and researchers to differentiate healthy and pathological gait patterns. Despite limitations like selection bias and cross-sectional design, the findings lay a foundation for future longitudinal studies and broader sampling. By incorporating wearable IMUs to capture natural, ’free-living’ gait, this study surpasses prior research focused on limited populations or controlled settings. These metrics offer valuable insights for early detection of gait abnormalities, advancing the clinical and research applications of spatiotemporal gait analysis.

## Figures and Tables

**Table 2:** Summary of participants and their demographic data.

		Sex		Height (cm)	Weight (Kg)	BMI	Smoking	Diabetes	Cholesterol	Hypertension	Any Falls in the Last 12 Months?	Any Problems with Balance?
		Male	Female	Mean (SD)	Mean (SD)	Mean (SD)	Yes	Yes	Yes	Yes	Yes	Yes
		Count (%)	Count (%)				Count (%)	Count (%)	Count (%)	Count (%)	Count (%)	Count (%)
Age category	(18–20)	15 (8.8%)	11 (7.5%)	171.56 (11.79)	67.54 (16.31)	18.02 (10.23)	5 (12.2%)	0 (0.0%)	0 (0.0%)	0 (0.0%)	2 (28.6%)	0 (0.0%)
	(21–30)	42 (24.6%)	43 (29.5%)	170.65 (8.89)	71.16 (14.83)	22.03 (4.64)	13 (31.7%)	2 (25.0%)	1 (4.5%)	0 (0.0%)	1 (14.3%)	4 (36.4%)
	(31–40)	41 (24.0%)	34 (23.3%)	170.13 (11.07)	73.85 (17.36)	23.88 (5.03)	11 (26.8%)	0 (0.0%)	4 (18.2%)	1 (6.3%)	2 (28.6%)	2 (18.2%)
	(41–50)	34 (19.9%)	26 (17.8%)	169.52 (10.85)	75.29 (16.58)	25.27 (5.35)	4 (9.8%)	5 (62.5%)	10 (45.5%)	5 (31.3%)	0 (0.0%)	0 (0.0%)
	(51–60)	26 (15.2%)	18 (12.3%)	170.34 (9.65)	79.41 (14.24)	25.73 (4.73)	5 (12.2%)	1 (12.5%)	5 (22.7%)	5 (31.3%)	2 (28.6%)	3 (27.3%)
	(61–70)	6 (3.5%)	11 (7.5%)	163.26 (10.69)	71.86 (13.09)	27.23 (5.56)	3 (7.3%)	0 (0.0%)	2 (9.1%)	4 (25.0%)	0 (0.0%)	0 (0.0%)
	(71–80)	3 (1.8%)	3 (2.1%)	163.71 (4.36)	72.30 (8.32)	27.10 (4.08)	0 (0.0%)	0 (0.0%)	0 (0.0%)	1 (6.3%)	0 (0.0%)	0 (0.0%)

**Table 3:** Spatiotemporal metric trends by age.

	18–20		21–30		31–40		41–50		51–60		61–70		71–80	
	Mean	SD	Mean	SD	Mean	SD	Mean	SD	Mean	SD	Mean	SD	Mean	SD
Cadence	105.83	8.02	108.40	9.17	111.87	8.86	111.75	9.24	110.05	9.47	115.91	7.85	111.05	12.49
Step length	0.70	0.12	0.73	0.14	0.72	0.17	0.72	0.14	0.71	0.17	0.67	0.15	0.60	0.19
Gait speed	1.21	0.19	1.30	0.25	1.34	0.28	1.35	0.23	1.32	0.31	1.32	0.25	1.16	0.30

**Table 4:** Spatiotemporal metric trends by age and gender (females).

	Female													
	18–20		21–30		31–40		41–50		51–60		61–70		71–80	
	Mean	SD	Mean	SD	Mean	SD	Mean	SD	Mean	SD	Mean	SD	Mean	SD
Cadence	109.68	9.95	111.88	9.25	114.41	9.45	115.14	8.75	114.35	8.36	118.87	8.21	111.03	17.04
Step length	0.63	0.10	0.71	0.15	0.64	0.15	0.63	0.11	0.66	0.16	0.58	0.09	0.48	0.14
Gait speed	1.17	0.22	1.32	0.25	1.24	0.26	1.28	0.25	1.29	0.29	1.23	0.20	0.99	0.27

**Table 5:** Spatiotemporal metric trends by age and gender (males).

	Male													
	18–20		21–30		31–40		41–50		51–60		61–70		71–80	
	Mean	SD	Mean	SD	Mean	SD	Mean	SD	Mean	SD	Mean	SD	Mean	SD
Cadence	103.42	5.63	105.22	7.77	109.76	7.86	109.26	8.91	107.08	9.19	110.99	4.16	111.08	9.99
Step length	0.74	0.11	0.75	0.14	0.80	0.15	0.79	0.12	0.75	0.17	0.80	0.15	0.71	0.17
Gait speed	1.23	0.16	1.30	0.26	1.42	0.26	1.41	0.21	1.34	0.32	1.47	0.27	1.32	0.28

**Table 6:** Spatiotemporal metric trends by height.

	Height (cm)											
	141–150		151–160		161–170		171–180		181–190		191–200	
	Mean	SD	Mean	SD	Mean	SD	Mean	SD	Mean	SD	Mean	SD
Cadence	116.54	6.36	114.96	9.25	111.38	9.34	108.59	8.28	104.65	7.61	103.52	5.04
Step length	0.62	0.06	0.64	0.15	0.67	0.15	0.77	0.12	0.79	0.17	0.91	0.05
Gait speed	1.23	0.11	1.27	0.27	1.26	0.27	1.37	0.25	1.35	0.31	1.49	0.12

**Table 7:** Spatiotemporal metric trends by height and gender (females).

	Female											
	Height (cm)											
	141–150		151–160		161–170		171–180		181–190		191–200	
	Mean	SD	Mean	SD	Mean	SD	Mean	SD	Mean	SD	Mean	SD
Cadence	116.76	6.94	115.87	9.41	112.02	9.29	109.45	7.39	112.47	6.28	116.76	6.95
Step length	0.60	0.04	0.63	0.15	0.64	0.13	0.80	0.10	0.77	0.11	0.60	0.05
Gait speed	1.20	0.09	1.26	0.27	1.23	0.25	1.43	0.19	1.43	0.08	1.21	0.10

**Table 8:** Spatiotemporal metric trends by height and gender (males).

	Male											
	Height (cm)											
	141–150		151–160		161–170		171–180		181–190		191–200	
	Mean	SD	Mean	SD	Mean	SD	Mean	SD	Mean	SD	Mean	SD
Cadence	115.22	0.00	108.54	4.31	110.06	9.48	108.58	8.41	104.28	7.52	103.52	5.04
Step length	0.74	0.00	0.71	0.16	0.72	0.18	0.77	0.13	0.79	0.18	0.91	0.05
Gait speed	1.40	0.00	1.28	0.25	1.32	0.30	1.36	0.25	1.34	0.32	1.49	0.12

**Figure A1:**
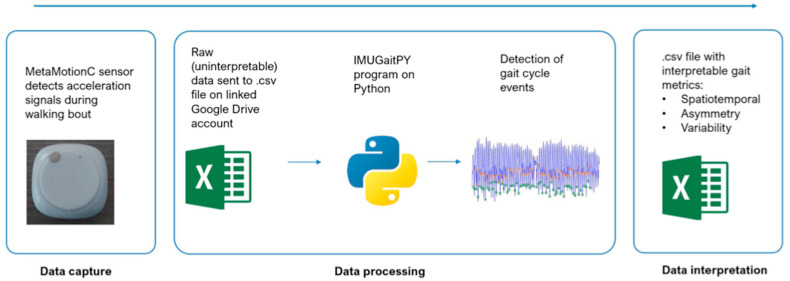
Flowchart describing the data processing workflow. MetaMotionC detects raw acceleration, gyroscope, and magnetometer signals, which are interpreted by a Python script known as the IMUGaitPy program. This is used to extract spatiotemporal gait metrics from the raw data and is obtained as a .csv file. Asymmetry and variability metrics can also be computed, and information regarding these derivations is detailed within the text. Figure taken from Natarajan et al. [49].

## Data Availability

The original contributions presented in the study are included in the article, further inquiries can be directed to the corresponding author.
